# Lithium Niobate Electro-Optic Modulation Device without an Overlay Layer Based on Bound States in the Continuum

**DOI:** 10.3390/mi15040516

**Published:** 2024-04-12

**Authors:** Guangyuan Chen, Ning Xue, Zhimei Qi, Weichao Ma, Wangzhe Li, Zhenhu Jin, Jiamin Chen

**Affiliations:** 1 State Key Laboratory of Transducer Technology, Aerospace Information Research Institute (AIR), Chinese Academy of Sciences, Beijing 100190, China; chengy@aircas.ac.cn (G.C.); xuening@mail.ie.ac.cn (N.X.); zhimei-qi@mail.ie.ac.cn (Z.Q.); mawc01@aircas.ac.cn (W.M.); liwz@aircas.ac.cn (W.L.); jinzhenhu@aircas.ac.cn (Z.J.); 2 School of Electronic, Electrical and Communication Engineering, University of Chinese Academy of Sciences, Beijing 100049, China

**Keywords:** thin-film lithium niobate, bound states in the continuum, lithium niobate electro-optic modulator

## Abstract

Electro-optic modulation devices are essential components in the field of integrated optical chips. High-speed, low-loss electro-optic modulation devices represent a key focus for future developments in integrated optical chip technology, and they have seen significant advancements in both commercial and laboratory settings in recent years. Current electro-optic modulation devices typically employ architectures based on thin-film lithium niobate (TFLN), traveling-wave electrodes, and impedance-matching layers, which still suffer from transmission losses and overall design limitations. In this paper, we demonstrate a lithium niobate electro-optic modulation device based on bound states in the continuum, featuring a non-overlay structure. This device exhibits a transmission loss of approximately 1.3 dB/cm, a modulation bandwidth of up to 9.2 GHz, and a minimum half-wave voltage of only 3.3 V.

## 1. Introduction

In recent years, due to the rapid development in the field of optical communications, various optical communication devices have emerged continuously, including communication wavelength lasers [1], optical isolators [2], optical resonant cavities [3], multi-frequency optical combs [4], and electro-optic modulators [5,6,7,8]. Among these, electro-optic modulators refer to devices that utilize the electro-optic effect to modulate light, with the process of encoding information onto a laser beam referred to as modulation. The device responsible for this process is called a modulator, with the laser acting as the carrier. Electro-optic modulator devices can be categorized based on their operating principles into acousto-optic modulators [9], thermo-optic modulators [10], and electro-optic modulators [5,6,7,8]. These devices, known for their high integration [11], high modulation rates [12], large modulation bandwidths [13], and excellent non-optical-to-optical compatibility [14], find wide applications in fields such as optical communications [15], high-speed modulation, and time-domain signal conversion [16].

Traditional electro-optic modulator devices typically consist of optical waveguides, traveling-wave electrodes or microstrip lines, a covering layer that matches the optical and microwave propagation speeds, and a substrate. The principle of electro-optic modulators relies on the property of optical waveguides made from electro-optic crystals (such as KDP-type crystals and lithium niobate) to change their refractive index in the presence of an external electric field. RF signals are applied to both sides of the optical waveguide (or on the top and bottom in the case of microstrip lines) to achieve optical modulation. The optical waveguide is used for the transmission of planar optical waves, while the traveling-wave electrodes or microstrip lines are employed to load RF signals generated by a signal source or voltage source on both sides of the optical waveguide. The covering layer is responsible for matching the propagation speeds of optical and microwave signals. However, the traditional optical waveguide design places high demands on the covering layer, requiring precise matching of materials, refractive indices, and thicknesses. Additionally, the structural design of the traveling-wave electrodes presents significant challenges, prompting researchers to continuously explore new structures for electro-optic modulator devices.

**Figure 1:**
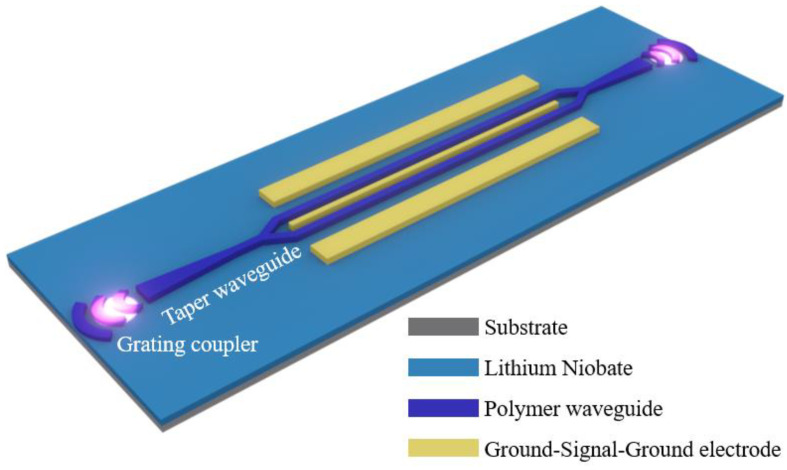
Electro-optic modulator with organic polymer + lithium-niobate-on-insulator (LNOI) substrate and no covering layer.

Bound states in the continuum (BICs) [17] are a unique optical resonance effect, ideally characterized by the absence of leaky modes. BICs have found numerous applications in photonic crystals [18], resonance enhancement [19], and optical waveguides [20]. Especially in optical waveguides, the absence of lateral leaky modes effectively reduces the complexity of optical waveguide structures and optical wave transmission losses [21]. By leveraging the advantages of BICs, we have designed a traveling-wave electrode electro-optic modulator device based on an organic polymer + lithium-niobate-on-insulator (LNOI) substrate, as shown in Figure 1. This device utilizes BICs in the waveguide to achieve ultra-low transmission losses without the need for a covering layer. Its transmission loss is approximately 1.3 dB/cm, the modulation bandwidth can reach up to 9.2 GHz, and the minimum half-wave voltage is only 3.3 V. This offers a new approach for the field of advanced integrated photonics.

## 2. Principle of Bound States in the Continuum in Optical Waveguides

Before we proceed to introduce the prepared electro-optic modulation devices, it is necessary to provide an introduction to the bound states in the continuum (BICs) and waveguide. Bound states in the continuum (BICs) are a fascinating optical phenomenon that have garnered widespread research interest in the fields of optics and electromagnetics. The BICs are known as embedded trapped modes, which correspond to discrete eigenvalues coexisting with extended modes of a continuous spectrum. Usually, energy is confined by low-energy potential wells, but energy higher than the potential well can exist in the form of continuous modes or resonant modes (leakage modes). Bound states in the continuum (BICs) exhibit the anomalous phenomenon where high-energy states in the continuous spectrum are still confined by low-energy potential wells. BICs refer to a specific class of modes existing in the continuous spectrum, characterized by frequencies distinct from the surrounding radiation modes while remaining confined within the system, not radiating energy to the far field. This unique waveguide phenomenon was initially proposed by Russian physicist Lev P. Rudnev in 1984 and has since been extensively investigated in various waveguide structures and optical systems. The formation of BICs is often closely associated with structural symmetries and phase-matching factors. In waveguide structures, the occurrence of BICs can be achieved by adjusting structural parameters such as the waveguide width, height, or medium refractive index. Unlike conventional waveguide modes, BICs exist in the continuous spectrum, with frequencies differing from the scattering coupling points generated by radiation modes. This allows for the realization of BICs in various waveguides and resonators, providing novel avenues for the design and control of optical devices. The study of optical continuous-spectrum-confined states involves intricate theoretical models and experimental techniques. Theoretical frameworks, rooted in the principles of wave optics and quantum mechanics, provide insights into the behavior of light within confined structures. These models guide experimentalists in designing and fabricating optical components capable of supporting continuous-spectrum-confined states. In practical terms, the implementation of continuous spectrum confinement often involves the use of advanced optical materials, metamaterials, and photonic crystal structures. These materials enable researchers to engineer specific optical properties and manipulate the dispersion of light, facilitating the creation of tailored environments for continuous spectrum confinement. The applications of optical continuous-spectrum-confined states are diverse and transformative. In telecommunications, for instance, the ability to control and manipulate a broad range of frequencies opens avenues for increased bandwidth and data transmission rates. Similarly, in sensing technologies, the enhanced sensitivity afforded by continuous spectrum confinement can revolutionize the detection of various physical and chemical parameters. Moreover, the exploration of continuous-spectrum-confined states in the realm of quantum optics holds promise for the development of quantum information processing and communication devices. The unique characteristics of these states, such as entanglement and superposition, present new opportunities for advancing quantum technologies. In conclusion, the study of optical continuous-spectrum-confined states represents a captivating journey into the manipulation of light at the fundamental level. This introduction provides a glimpse into the theoretical foundations, experimental methodologies, and potential applications of this emerging field. As researchers delve deeper into the intricacies of continuous spectrum confinement, we can anticipate groundbreaking discoveries and technological advancements that will shape the future of optics and photonics.

Integrated optics involves the integration of optical components and devices on a single substrate, typically made of semiconductor materials such as silicon or III-V compounds. The core idea is to confine and manipulate light within these integrated structures, allowing for the seamless integration of various optical functions. The basic building blocks of integrated optics include waveguides, couplers, modulators, detectors, and other passive and active devices. Waveguides, often in the form of thin channels, guide light signals through the substrate, ensuring minimal loss and efficient transmission. Couplers facilitate the interaction between different waveguides, enabling the creation of complex optical circuits. The interaction between light and the semiconductor material in integrated optics is governed by principles of waveguide theory, interference, and diffraction. The miniaturization of optical components on the chip scale is made possible by leveraging semiconductor fabrication techniques that have been honed in the microelectronics industry. Integrated optics finds application in various fields, with telecommunications being a primary beneficiary. Optical communication systems benefit from compact and efficient integrated devices, leading to enhanced data transmission rates and reduced power consumption. Photonic integrated circuits (PICs) have become integral components in optical networks, facilitating the processing and routing of optical signals. Sensing technologies also leverage integrated optics for enhanced performance. Miniaturized sensors based on integrated optical devices offer improved sensitivity, enabling the detection of minute changes in environmental parameters. This has applications in environmental monitoring, healthcare, and industrial sensing. The field of biophotonics has witnessed significant advancements through integrated optics. Lab-on-a-chip devices integrate optical components for applications such as biosensing, DNA sequencing, and cellular imaging. The compact nature of these devices enhances portability and ease of use in various analytical and diagnostic applications. In the other hand, optical waveguides play a pivotal role in modern photonics, serving as essential components for guiding and manipulating light in a variety of applications. These waveguides form the backbone of diverse technologies, from telecommunications to sensors, lasers, and integrated optical circuits. This introduction provides an overview of the background and significance of optical waveguides. Optical waveguides are structures designed to confine and guide light, preventing its dispersion and ensuring efficient transmission along a predefined path. They exploit the principles of total internal reflection, where light waves are confined within a high-refractive-index medium, minimizing energy loss and allowing for controlled propagation. This confinement enables the creation of compact and efficient optical devices. The fundamental concept of waveguiding dates back to the early 20th century, with significant advancements in the mid-20th century. The advent of fiber optics revolutionized long-distance communication, replacing traditional copper cables with optical fibers. These fibers, acting as waveguides, allowed for the transmission of vast amounts of data over long distances with minimal signal loss. Planar waveguides, another crucial development, involve thin films or layers of high-refractive-index material deposited on substrates. These waveguides find applications in integrated optics, where miniaturization and integration are essential. Silicon-based waveguides, for instance, have become prominent in photonic integrated circuits due to their compatibility with existing semiconductor technologies. The design and engineering of optical waveguides have evolved significantly to meet the demands of various applications. Different types of waveguides, including single-mode and multimode waveguides, have been developed to accommodate specific requirements. Single-mode waveguides, with a narrow core, are suitable for high-speed, long-distance communication, while multimode waveguides, with a larger core, are employed in short-distance communication and optical sensing. Recent developments in materials science and nanotechnology have further expanded the capabilities of optical waveguides. Metamaterials and photonic crystals, engineered to control the flow of light at the subwavelength scale, offer unprecedented opportunities for tailoring waveguide properties. Additionally, advancements in nonlinear optics and plasmonics have enabled the development of waveguides with enhanced functionalities, such as frequency conversion and sensing. In conclusion, optical waveguides have become integral to numerous technologies, underpinning the advancements in telecommunications, sensing, and integrated photonics. The continuous exploration of novel materials and fabrication techniques ensures that optical waveguides will continue to play a central role in shaping the future of optical communication and photonics applications.

## 3. Theoretical Modeling, Results, and Discussion

**Figure 2:**
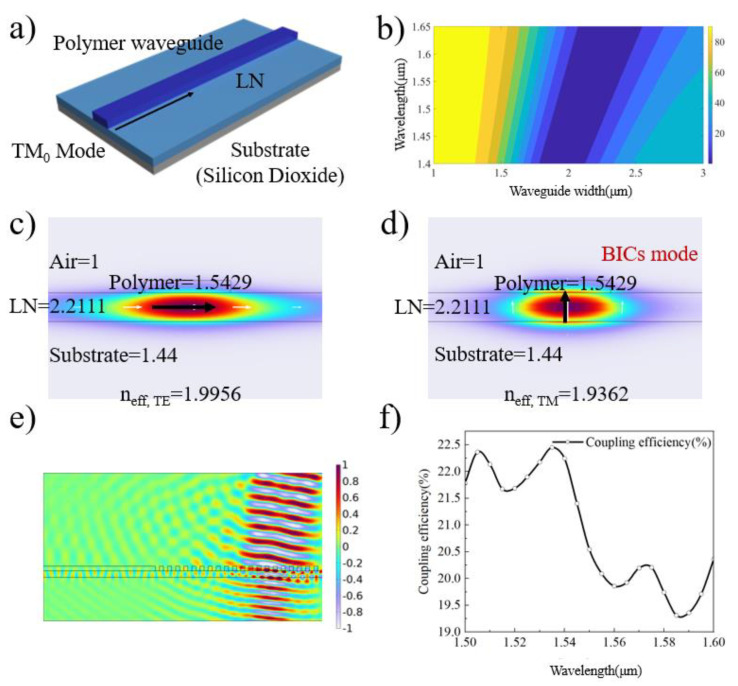
(**a**) Schematic illustration of a ridge waveguide composed of polymer, LN, and substrate. The height of the polymer waveguide is 400 nm. (**b**) Transmission losses at different optical wavelengths and polymer waveguide widths within the TM_0_ mode. When the polymer waveguide width is approximately 2.1 μm and the optical wavelength is 1550 nm, the corresponding transmission loss is close to zero (without the metal electrode). The color bar represents the magnitude of transmission loss, the unit of which is dB/cm. (**c**,**d**) Mode distribution maps for the TE_0_ and TM_0_ modes. In these figures, the polymer height is 500 nm, its width is 2.1 μm, and the LN height is 700 nm. White arrows indicate the direction of the electric field component. (**e**) Coupling simulation field distribution for the grating structure at 1550 nm. The grating width is 435 nm, the duty cycle is 50%, and the incident angle is 9.4 degrees. (**f**) Coupling efficiency of the same grating structure at different wavelengths. The direction is from free space to the waveguide. The coupling efficiency is calculated by normalizing the energy from the right upper corner (E_input_) and the energy in the left-side waveguide layer (E_out_) to mitigate the effects of size.

First, we designed the prototype of the electro-optic modulator based on the lithium-niobate-on-insulator (LNOI) substrate–ridge waveguide. We designed an optical waveguide based on BICs that exhibits ultra-low transmission losses in the TM fundamental mode. The three-dimensional structure of the waveguide is shown in Figure 2a, consisting from top to bottom of the electron beam resist, lithium niobate (LN), and the substrate. When the optical field enters the optical waveguide, due to the difference in intrinsic refractive indices (where that of the polymer (n_pol_ = 1.5429) is lower than that of LN (n_e_ = 2.1376, n_o_ = 2.2111)), the optical field propagates along the LN. Using a polymer as a cover layer for the waveguide offers the advantage of significantly simplifying fabrication, avoiding the roughness introduced by LN etching processes that can further impact transmission losses.

BICs in the optical waveguide refer to the occurrence of the TM_0_ mode within the continuous TE modes. Typically, the TE_0_ and TM_0_ modes are more likely to appear in regions with a high effective refractive index (low-energy potential wells), and BICs in the optical waveguide can be achieved by designing the structural parameters of the waveguide appropriately. To achieve BICs in a heterogeneous-structure waveguide, it is essential to tailor the structural parameters of the covering layer waveguide, especially its width [21]. We calculated the transmission losses of the optical waveguide at different widths using numerical simulation software, as shown in Figure 2b. The defined transmission loss refers to the ratio of the energy of the optical waveguide not confined within the width of the polymer optical waveguide to the total optical field energy in the LN layer. When the polymer waveguide width is approximately 2.1 μm at a wavelength of 1550 nm, the corresponding transmission loss approaches zero. This implies that we can precisely control the transmission loss of the optical field by controlling the width of the polymer waveguide. Furthermore, we used finite element simulation software to study the mode distribution of the optical field in the three-layer waveguide structure, including the TE_0_ and TM_0_ modes, as depicted in Figure 2c,d. The effective refractive index values for the TE_0_ mode (n_eff, TE_ = 1.9956) and TM_0_ mode (n_eff, TM_ = 1.9362) correspond to the energy well size. However, different mode distributions are strictly confined within the LN layer and the waveguide width range.

**Figure 3:**
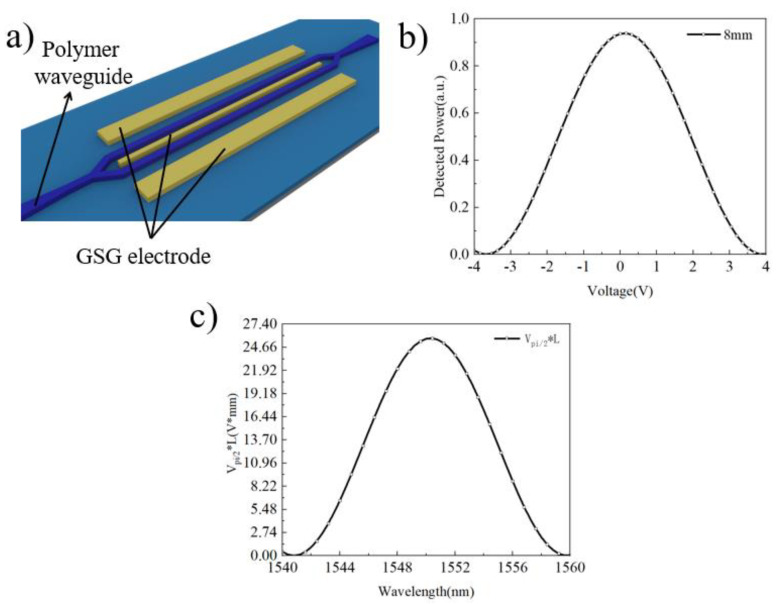
(**a**) Schematic illustration of the GSG electrode distribution. (**b**) Optical field transmission curves under different applied voltages for the GSG electrode. The GSG electrode was simulated using gold as the material, with a height of 120 nm and a separation of 4 μm from the polymer waveguide. The half-wave voltage is approximately 3.8 V, and the full-wave voltage is approximately 7.5 V. The electro-optic interaction length is 8 mm. (**c**) The V_π/2_·L versus wavelength curve for wavelengths from 1540 nm to 1560 nm; when the wavelength is 1550 nm, the maximum V_π/2_*L is obtained, at 23.74 V*mm.

In addition to the straight optical waveguide, electro-optic modulation devices include two other components: free-space-to-chip coupling and traveling-wave electrodes (Ground–Signal–Ground, GSG electrode). We selected a grating as the free-space-to-chip coupling component, which offers advantages such as a tunable coupling wavelength and good transition. The design efficiency of the grating structure at 1550 nm was simulated using finite element simulation software, and the coupling mode distribution and efficiency are shown in Figure 2e,f. Figure 2e illustrates the distribution of the optical field (bare optical fiber) when it enters the grating structure from the upper right corner. The propagation of the optical field to the left confirms that the designed grating structure can introduce and emit the optical field. Figure 2f presents the coupling efficiency of the same grating structure at different optical wavelengths, indicating that the coupling efficiency is highest at around 1540 nm, reaching approximately 22.5%. Furthermore, since the electro-optic modulation device utilizes the electro-optic effect of the LN crystal, we studied the impact of the GSG electrode on the performance of the electro-optic modulator. Considering that the transmission mode of the optical field is the TM_0_ mode, and to maximize the utilization, which is the electro-optic effect of LN, we selected the γ_51_ component from the electro-optic coefficient tensor (γ_51_ = 27 pm/V) [20]. As shown in Figure 3a,b, the optical field transmission curves under different applied voltages for the GSG electrode are provided, and the half-wave voltage is approximately 3.8 V. Figure 3b presents the simulation results from finite element analysis software. The results in the figure are intended solely to illustrate the variations in optical power under different DC biases, demonstrating that the applied voltage can participate in the modulation of optical field energy.

In addition, we have provided V_π/2_·L in the theoretical calculation section, with a diagram showing the relationship between V_π/2_·L and the wavelength. We believe that the electro-optic coefficient of TFLN is non-dispersive, so V_π/2_·L is only related to the transmission loss, as shown in Figure 3c. The calculation of V_π/2_·L in Figure 3c is usually linear, but in BIC-based electro-optic modulation devices, voltage fluctuations can affect the refractive index change of TFLN, thereby changing the BICs’ conditions, increasing transmission losses, and affecting the relationship with changes in V_π/2_·L, resulting in parabolic and non-conformal effects. 

**Figure 4:**
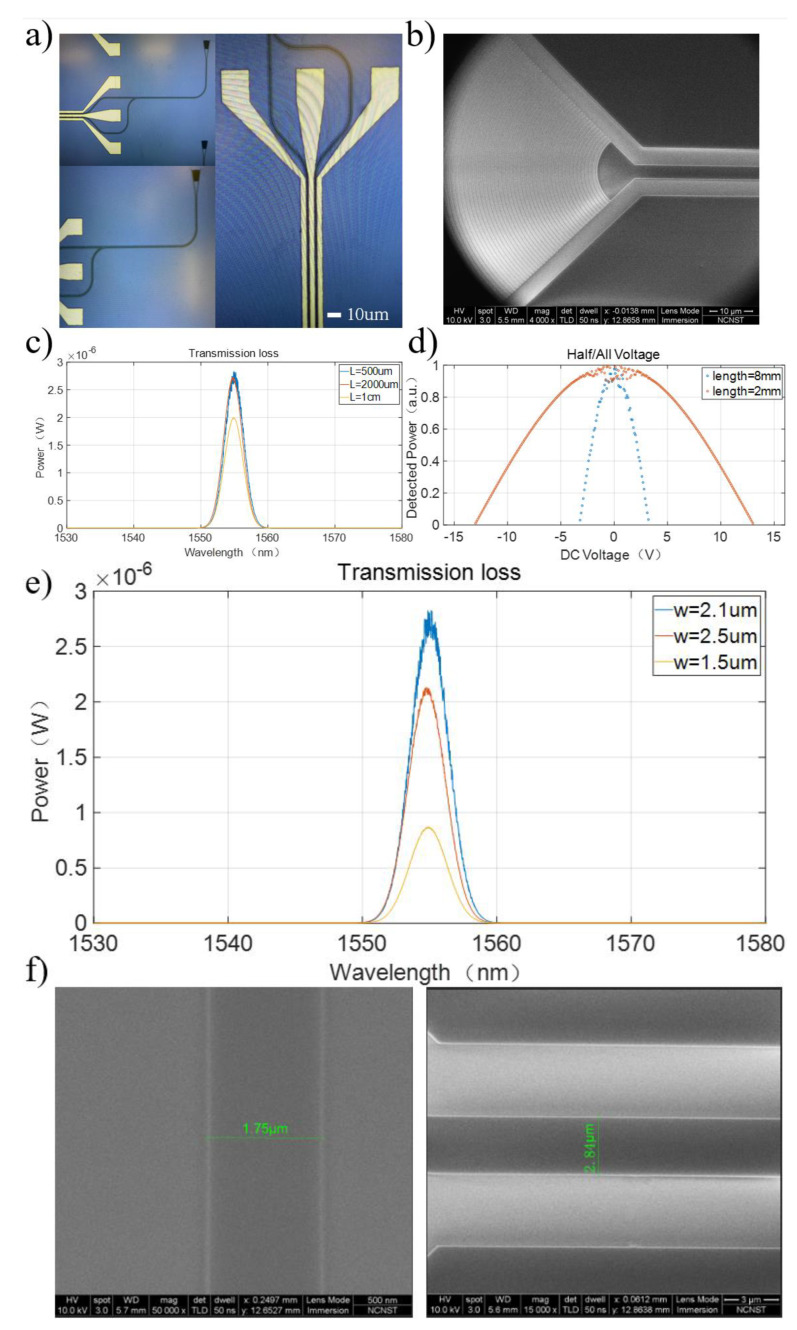
(**a**) Optical microscope image of a single LN electro-optic modulator device. The actual processed size of waveguide is ~0.3 µm wider than the size we designed, which is 2.4 μm. (**b**) SEM image of the grating section of the LN electro-optic modulator device. (**c**) Transmission losses at different optical waveguide lengths. The optical fiber input energy is approximately 1 mW, and the optical fiber energy at the receiving end is approximately 1 mW when the optical waveguide length is 500 μm. The calculated transmission loss is approximately 1.3 dB/cm. (**d**) Half-wave voltage curves for different electro-optic interaction lengths. The inner curve represents a device with an 8 mm electro-optic interaction length, with a corresponding half-wave voltage of 3.3 V. The external curve represents a device with a 2 mm electro-optic interaction length, with a corresponding half-wave voltage of 12.4 V. (**e**) The transmission losses under different polymer waveguide widths are shown in the graph. The blue line corresponds to a polymer waveguide width of 2.1 μm, with a transmission loss of 1.3 dB/cm. The red line corresponds to a polymer waveguide width of 2.5 μm, with a transmission loss of 3.2 dB/cm. The orange line corresponds to a polymer waveguide width of 1.5 μm, with a transmission loss of 4.8 dB/cm. The laser source we employed was a non-tunable laser, specifically the Rainbow 1550 Pro model from NPI Lasers. (**f**) SEM image of the different waveguide widths of 1.5 μm and 2.5 μm.

Based on the simulation results mentioned earlier, we fabricated a balanced electro-optic modulator based on LNOI. Firstly, we selected a commercial LNOI wafer as the substrate for the modulator. The bottom layer of the wafer is made of silicon, the middle layer is thermally oxidized silicon dioxide with a thickness of 2 μm, and the top layer is LN with a thickness of 700 nm. Considering LN’s electro-optic properties, the crystal was X-cut to maximize the required electro-optic coefficient. This LNOI wafer structure ensures that the LN crystal serves as the substrate for the electro-optic modulator. The next step involved creating a polymer optical waveguide on the LN wafer surface. Based on the simulation results, we chose the electron beam resist ZEP520A as the polymer needed for the optical waveguide, with an intrinsic refractive index of 1.5429 (λ@1550 nm), and the thickness of the polymer layer was about 440 nm. Using an electron beam resist as the optical waveguide material, as is common in electron-beam lithography, ensures ease of access and simplifies the fabrication process. As mentioned in the second section, using a photoresist as the optical waveguide material avoids sidewall losses and roughness effects on transmission losses caused by dry etching. During the experiment, the commercial LN wafer was sliced to 1.5 cm × 1.5 cm, cleaned, and then uniformly coated with electron beam resist. Electron-beam lithography (EBL) was employed for the graphical representation of optical waveguide structures, while the patterning of electrode structures was achieved through ultraviolet photolithography, followed by metal deposition and lift-off processes to manufacture the electro-optic modulator structure. The EBL dose was 230 μc/cm^2^, and amyl acetate was chosen for the development solution. Figure 4a shows an optical microscope image of a single device, including the grating, tapered optical waveguide, electro-optic interaction region, and GSG electrode section. Additionally, we observed and characterized certain structural parameters of the device under a scanning electron microscope (SEM) to ensure sample consistency with the design, as shown in Figure 4b.

To validate the performance of the electro-optic modulator, we initially tested the transmission losses of the optical waveguide. The mode transmitted in the optical waveguide is the TM_0_ mode. The optical field in the optical waveguide was introduced and extracted using fiber-to-grating coupling, and we fabricated straight optical waveguides of lengths 500 μm, 2000 μm, and 1 cm with all other parameters except the waveguide length being identical. The calculated minimum transmission loss of the optical waveguide was approximately 1.3 dB/cm, as shown in Figure 4c. The measured coupling efficiency was 17%, which still shows a discrepancy with our theoretical values, mainly attributed to errors in the photolithography process. As for the transmission loss inside the optical waveguide, the measured value was 1.3 dB/cm. With an increase in device length, this loss did not vary significantly, indicating the stability of BICs concerning variations in mode (or structural parameters).

We believe that the transmission loss in the actual test mainly comes from the actual processing error, which includes the surface roughness, structural size offset, and deterioration of electron beam resist materials in the actual environment. Next, we measured the half-wave/full-wave voltage of the electro-optic modulator using a DC testing platform, which included a communication band light source, fiber polarization controller, three-dimensional electric translation stage, DC signal generator, and optical power meter. In electro-optic modulation devices, the half-wave/full-wave voltage typically refers to the voltage required to achieve a half-wave or full-wave phase shift in the intensity of the output signal. In the experiment, we prepared electro-optic modulator devices with interaction lengths of 2 mm and 8 mm under consistent test conditions. We measured the relationship between the optical field output of the electro-optic modulator and the applied voltage, as shown in Figure 4d. The half-wave voltage for the 2 mm device was approximately 12.4 V, while for the 8 mm device, it was approximately 3.3 V.

Furthermore, for BICs, the width of the polymer waveguide is a crucial factor in determining the existence of BIC modes. We prepared polymer optical waveguides with a length of 500 μm: one with a width of 2.1 μm, matching the BICs, and the other with widths of 1.5 μm and 2.5 μm, deviating from the BICs. The transmission losses corresponding to the different waveguide widths were measured separately and are shown in Figure 4e. Under the same conditions, we found that the transmission loss of the 2.1 μm waveguide width was only 1.3 dB/cm, significantly lower than the transmission losses of 3.2 dB/cm for the 2.5 μm width and 4.8 dB/cm for the 1.5 μm width. Since the presence of BICs reduces the transmission loss of the waveguide, an increase in transmission loss under otherwise consistent external conditions indicates the disruption of the BICs. This suggests that variations in the waveguide width have a significant impact on BICs. Figure 4f displays local SEM images of the optical waveguides with designed dimensions of 1.5 μm and 2.5 μm in width. Due to fabrication tolerances, the actual measured dimensions were found to be 1.75 μm and 2.84 μm, respectively.

**Figure 5:**
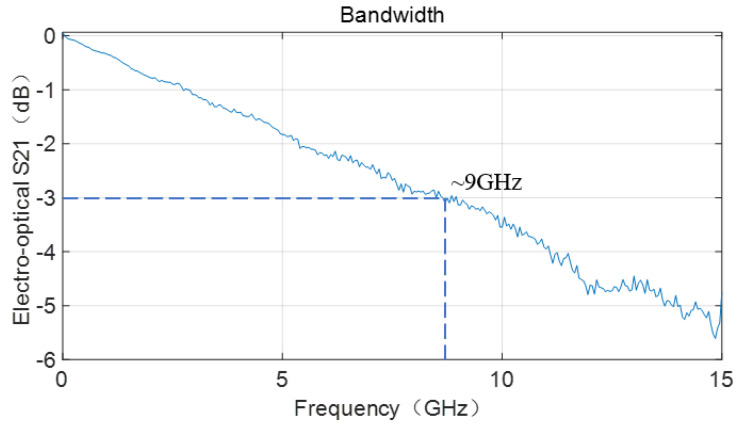
Bandwidth curve of the electro-optic modulator device, with a 3 dB bandwidth of approximately 9.2 GHz.

As mentioned earlier, due to the presence of BICs, the optical beam propagates in the LN layer within the polymer waveguide width with almost no transmission losses. The advantages mentioned above ensure that the optical transmission mode of this electro-optic modulator device is virtually unrestricted. As a non-overlay device, LN has excellent microwave transmission rates [22] that also ensure its suitability as the foundation for an electro-optic modulator. Considering the absence of impedance mismatches and transmission rate issues that can arise from surface covering layers, we tested the 3 dB bandwidth of the device, as shown in Figure 5. The relatively low bandwidth of electro-optic modulators in BIC mode is actually limited by the speed mismatch between microwaves and light waves. In this work, we removed the limitation of the cover layer in exchange for a simpler device structure and lower process complexity. However, the speed mismatch between the two was greater, with a bandwidth of only ~9.2 GHz. Although there is a gap when compared to commercial and laboratory-level electro-optic modulators in terms of bandwidth, there is still research value in the field of non-overlay electro-optic modulators.

Compared to traditional electro-optic modulation devices, although the transmission bandwidth and loss based on BICs have not yet been optimized (limited by processing technology), the existing lithium niobate electro-optic modulators can achieve a bandwidth of about ~110 GHz [23], and the transmission loss can also be as low as 6 dB/m [24]. However, our design still proposes a new path for the development of future electro-optic modulators.

Indeed, there have been related works on applying BICs to lithium niobate material systems. Table 1 summarizes the key parameters in comparison with the relevant literature in this field.

## 4. Summary

We have proposed a non-overlay electro-optic modulator based on LNOI, which takes advantage of gratings as fiber–chip coupling elements and leverages the benefits of BICs to achieve low transmission losses of 1.3 dB/cm in TM mode transmission. In terms of electrical performance, the device exhibits a half-wave voltage of approximately 3.3 V for an interaction length of 8 mm, corresponding to a −3 dB bandwidth of around 9 GHz. The research on the above electro-optic modulator device provides new directions for future chip integration and system complexity in electro-optic modulation, with the potential for applications in miniaturized photonics chips, compact photonic systems, and high-speed optical communication.

## Figures and Tables

**Table 1:** A comparison of our work and refs. [25,26].

	Transmission Loss	Characteristic Metrics	Taper Waveguide
Ref. [25]	<4.0 dB (insertion loss)	40 Gbps/channel (data transmission)	No
Ref. [26]	10 dB/cm	4.05% W^−1^cm^−2^ (conversion efficiency)	No
Our work	1.3 dB/cm	9.2 GHz (EO bandwidth)	Yes

## Data Availability

The original contributions presented in the study are included in the article, further inquiries can be directed to the corresponding author.
